# Scallop IKK1 Responds to Bacterial and Virus-Related Pathogen Stimulation and Interacts With MyD88 Adaptor of Toll-Like Receptor Pathway Signaling

**DOI:** 10.3389/fimmu.2022.869845

**Published:** 2022-03-29

**Authors:** Lingling Li, Wenjuan Liu, Nini Fan, Fangshu Li, Baoyu Huang, Qian Liu, Xiaomei Wang, Yanxin Zheng, Xiuxiu Sang, Juan Dong, Xiaona Wang, Lei Wei, Yaqiong Liu, Meiwei Zhang, Jilv Ma, Jiwen Chen, Yitao Qi, Xiaotong Wang

**Affiliations:** ^1^ School of Agriculture, Ludong University, Yantai, China; ^2^ Ocean School, Yantai University, Yantai, China; ^3^ Changdao Enhancement and Experiment Station, Chinese Academy of Fishery Sciences, Yantai, China; ^4^ College of Life Sciences, Shaanxi Normal University, Xi’an, China

**Keywords:** scallop, innate immunity, IKK, MyD88, IRF, signaling pathway

## Abstract

IKK proteins are key signaling molecules in the innate immune system of animals, and act downstream of pattern recognition receptors. However, research on IKKs in invertebrates, especially marine mollusks, remains scarce. In this study, we cloned CfIKK1 gene from the Zhikong scallop (*Chlamys farreri*) and studied its function and the signaling it mediates. The open reading frame of *CfIKK1* was 2190 bp and encoded 729 amino acids. Phylogenetic analysis showed that *CfIKK1* belonged to the invertebrate *IKKα/IKKβ* family. Quantitative real-time PCR analysis revealed the ubiquitous expression of *CfIKK1* mRNA in all scallop tissues and challenge with lipopolysaccharide, peptidoglycan, or poly(I:C) significantly upregulated the expression of *CfIKK1*. Co-immunoprecipitation assays confirmed the interaction of CfIKK1 with scallop MyD88 (Myeloid differentiation actor 88, the key adaptor of the TLR signaling pathway) *via* its N-terminal kinase domain. Additionally, CfIKK1 protein could form homodimers and even oligomers, with N-terminal kinase domain and C-terminal scaffold dimerization domain playing key roles in this process. Finally, the results of RNAi experiments showed that when the scallop IKK1 gene was suppressed, the expression of IRF genes also decreased significantly. In conclusion, CfIKK1 could respond to PAMPs challenge and interact with MyD88 protein of scallop TLR signaling, with the formation of CfIKK1 dimers or oligomers. At the same time, the results of RNAi experiments revealed the close regulatory relationship between *IKK1* and *IRF* genes of scallop. Therefore, as a key signal transduction molecule and immune activity regulator, CfIKK1 plays important roles in the innate immune system of scallops.

## 1 Introduction

Invertebrates, which are considered to lack the capability for eliciting adaptive or acquired immunity, have to rely on their innate immune system to resist infections by external pathogenic microorganisms (1). Innate immunity is triggered through the recognition of pathogen-associated molecular patterns (PAMPs) by pattern recognition receptors, such as Toll-like receptors (TLRs), which then through a series of signal transduction molecules finally activate transcription factors, such as interferon regulatory factors (IRFs) and nuclear factor-kappa B (NF-κB), to initiate the innate immune response for killing the invading pathogens (2, 3).

Inhibitor of NF-κB (IκB) kinases (IKKs) are essential signal transduction molecules in the innate immune system. They receive immune signals from the upstream regulators and eventually activate key transcription factors, such as NF-κB and IRFs (4, 5). Generally, two sets of *IKK* genes have been identified: the canonical genes *IKKα* and *IKKβ*, and the IKK-related genes TANK-binding kinase 1 (*TBK1*) and *IKKϵ* (4). IKKα and IKKβ, which were first molecularly cloned from a purified 900 kDa cytokine-induced IκB kinase complex, have been found to play important roles in IκB phosphorylation and NF-κB activation (6). The IKK complex comprises three proteins: IKKα, IKKβ, and IKKγ (7). IKKα and IKKβ carry out the catalytic functions, whereas IKKγ (also named the NF-κB essential modulator or NEMO) acts as a scaffold and regulatory subunit (8, 9). Generally, in the MyD88 (Myeloid differentiation actor 88)-dependent TLR pathway, the adaptor molecule MyD88 can recruit signaling proteins such as TRAF6 (TNF receptor associated factor 6) and IRAK (Interleukin-1-receptor associated kinase) proteins, which in turn transmit signals down and activates the IKK complex. Both IKKα and IKKβ can form homodimers, which is a crucial step for IκB phosphorylation and NF-κB activation (10, 11). TBK1 and IKKϵ, which were identified after IKKα and IKKβ had been cloned owing to the similarity of their sequences (12, 13), participate in innate immunity mainly by activating the transcription factors IRF3 and IRF7 in vertebrates (4, 14).

Whereas the roles of IKKs in mediating signal transduction and immune function in vertebrates have been reported in detail, there are relatively fewer studies on their activities in invertebrates. According to reports, the *Drosophila melanogaster* genome encodes homologous genes of the vertebrate IKK family. That is, immune-response deficient 5 (*IRD5*) and *Kenny* are the homologs of mammalian *IKKβ* and *IKKγ*, respectively (12). These two proteins can bind to each other to form a signaling complex, which would be crucial for the activation of antibacterial genes *via* the immune deficiency pathway (15). Other reports on the identification and functional verification of invertebrate *IKK* genes include those on three *IKK* genes from the disk abalone (16); two *IKKα/IKKβ* genes from the Pacific oyster (17, 18); *IKKβ*, *IKKϵ1*, and *IKKϵ2* from the mud crab (19); and *IKKβ* and *IKKϵ* from the Pacific white shrimp (20). These studies have confirmed that these invertebrate *IKK* genes can respond to pathogen stimuli and their PAMPs and participate in the host’s innate immune signal transduction and transcription factor (NF-κB) activation processes. Moreover, these studies revealed the conserved immune function of invertebrate IKKs. However, the details of the involvement of invertebrate IKKs in signal transduction and in the activation of antiviral or antibacterial cytokines are largely unknown and require further research to elucidate.

Marine mollusks are a good source of high-quality protein for human consumption. The Zhikong scallop (*Chlamys farreri*, a bivalve species), which is naturally distributed in the coastal areas of China, Japan, and Korea, has important commercial value in China (21). However, in the past two decades, the large-scale death of scallops owing to diseases has caused huge economic losses to—and seriously affected the healthy development of—the scallop farming industry (22, 23). Therefore, there is an urgent need to better understand the innate immune mechanisms of scallops so that new strategies for controlling their susceptibility to infectious diseases can be developed.

In view of this, researchers have devoted themselves to elucidating the innate immune mechanism of scallops and have achieved some results in this regard (24, 25). However, the exact details about the signal transduction and regulatory mechanisms of the scallop immune system remain unclear. In this study, the *IKK1* gene of *C. farreri* (*CfIKK1*) was cloned and characterized for the first time. The expression profiles of *CfIKK1* mRNA in different scallop tissues and in the hepatopancreas of individuals that had been challenged with different PAMPs were examined. Additionally, the immune signal transduction mediated by CfIKK1 was studied *via* co-IP and RNAi assays. Our research results add to the general knowledge about the immune mechanisms of invertebrates and lay the foundation for the development of future anti-disease strategies for scallops.

## 2 Materials and Methods

### 2.1 Animals, Immune Stimulation, and Sample Collection

The healthy adult Zhikong scallops used in this study, which were purchased from a local farm in Yantai (Shandong Province, China), had an average shell height of 55 mm. Before the study, the scallops were conditioned in filtered and aerated seawater at 19 ± 0.5°C for 10 days. After the acclimation period, 200 scallops were randomly divided into four groups of 50 individuals each. Through intramuscular injections, the animals in the first group each received 50 μL of phosphate-buffered saline (PBS: 0.14 M sodium chloride, 1.5 mM potassium phosphate monobasic, 3 mM potassium chloride, and 8 mM disodium hydrogen phosphate dodecahydrate; pH 7.4), those in the second group received 50 μL of lipopolysaccharide (LPS, 10 mg/mL in PBS; Sigma, USA), those in the third group received 50 μL of peptidoglycan (PGN, 10 mg/mL in PBS; Sigma), and those in the fourth group received 50 μL of polyinosinic–polycytidylic acid (poly(I:C), 1.0 mg/mL in PBS; *In vivo*Gen, USA). At 0, 6, 12, 24, 48, and 72 h after challenge, six scallops from each group were collected randomly, and the gills were gathered. Additionally, 10 types of tissue (hemocytes, smooth muscle, striated muscle, mantle, gill, hepatopancreas, feet, female gonad, male gonad, and kidney) were collected from six untreated scallops to investigate the bodily distribution of *CfIKK1* mRNA transcripts.

### 2.2 RNA Isolation and First-Strand cDNA Synthesis

TRIzol reagent (Invitrogen, USA) was used to isolate total RNA from the scallop tissues. First-strand cDNA synthesis was then carried out with the AG Evo M-MLV Plus cDNA Synthesis Kit (Accurate Biology, China), using DNase I-treated RNA as the template and an oligo(dT) adaptor as the primer. The reaction was performed at 42 °C for 30 min and terminated by heating at 95°C for 5 min.

### 2.3 *CfIKK1* Sequence Cloning and Analysis

Through sequence alignment studies, several *IKK* genes were found in the *C. farreri* genome (26). For this present study, we focused on one *IKK* gene and designed CfIKK1-F and CfIKK1-R (**Table 1**) primers for the PCR amplification of its open reading frame (ORF). The PCR product was purified and cloned into the pEASY-T1 vector (TransGen Biotech, China), and the recombinant plasmid was then used to transform competent cells. Positive clones were sent to Personalbio Technology (China) for gene sequencing.

**Table 1:** Primers used in this research.

Primers	Sequence (5’-3’)	Application
CfIKK1-F	ATGATTCAACAGAGCAACACTC	ORF cloning
CfIKK1-R	TCATGTGTTCGCCAACGGTATT	ORF cloning
CfIIKK1-qRT-F	AAATGGGAACCTTACTGACTCT	qRT-PCR
CfIKK1-qRT-R	CTGCTGGTCAAGTCTATTACAT	qRT-PCR
CfIIKK2-qRT-F	GTGCCACGGGCGATGTATTT	qRT-PCR
CfIKK2-qRT-R	TTCTCGCAGAGTTCCATAACAA	qRT-PCR
CfIIKK3-qRT-F	AGGAAAATGATTCGTTAGACCG	qRT-PCR
CfIIKK3-qRT-R	GTATCCCTATCAATGCCGTTA	qRT-PCR
CfRel-qRT-F	ACTTGCCGTCCGATGTGATGAG	qRT-PCR
CfRel-qRT-R	GGGACCCTTGTGAGCCGAG	qRT-PCR
CfIκB1-qRT-F	AGGACGAAGAAGGGGATACG	qRT-PCR
CfIκB1-qRT-R	ACTGCAAGGTGAAGGGGTGT	qRT-PCR
CfIκB2-qRT-F	GCGTTACATCTCGCTGTTTTG	qRT-PCR
CfIκB2-qRT-R	TCCATATCACCCTGTCGGCA	qRT-PCR
CfIRF1-qRT-F	GAGAGTGAAGCCGACAGTATG	qRT-PCR
CfIRF1-qRT-R	CAATGTGGTAGTCTGGGCAAAT	qRT-PCR
CfIRF2-qRT-F	GGGAACCACAAGGGGCAACA	qRT-PCR
CfIRF2-qRT-R	CCGCTATCCGCATCTGACTC	qRT-PCR
CfIRF3-qRT-F	AATACAATACCTGGGCTTACCT	qRT-PCR
CfIRF3-qRT-R	GCAGTGTCAACTCCCTCTCG	qRT-PCR
EF-1α-qRT-F	GCCATACCGCTCACATTGCT	qRT-PCR
EF-1α-qRT-R	CCAGAACGACGGTCGAGTTT	qRT-PCR
CfIKK1-FL-flag-F	CTCCATATGACTAGTCTCGAGATGATTCAACAGAGCAACACTC	Protein expression
CfIKK1-FL-flag-R	TACCACGCGTGAATTCTCGAGTCATGTGTTCGCCAACGGTATT	Protein expression
CfIKK1-P1-flag-R	TACCACGCGTGAATTCTCGAGGTTCAGCATGGAATCTAGCACA	Protein expression
CfIKK1-P2-flag-R	TACCACGCGTGAATTCTCGAGAAACAGGAATACCACCCAGTCT	Protein expression
CfIKK1-P3-flag-F	CTCCATATGACTAGTCTCGAGAAGAAAGGCGGATCTTCGGAGA	Protein expression
CfMyD88-myc-F	CATGGAGGCCCGAATTATGGCAATGGCGGATATCGA	Protein expression
CfMyD88-myc-R	CTCGGTCGACCGAATTTTACTCTCCTCGTTTTTTATTTTT	Protein expression
CfIKK1-FL-myc-F	CATGGAGGCCCGAATTATGATTCAACAGAGCAACACTC	Protein expression
CfIKK1-FL-myc-R	CTCGGTCGACCGAATTTCATGTGTTCGCCAACGGTATT	Protein expression
CfIKK1-P1-myc-R	CTCGGTCGACCGAATTGTTCAGCATGGAATCTAGCACA	Protein expression
CfIKK1-P3-myc-F	CATGGAGGCCCGAATTAAGAAAGGCGGATCTTCGGAGA	Protein expression
CfIKK1-sense	GGACCUCAGAAAGGUAUUATT	RNAi
CfIKK1-anti-sense	UAAUACCUUUCUGAGGUCCTT	RNAi
NC-sense	UUCUCCGAACGUGUCACGUTT	RNAi
NC- anti-sense	ACGUGACACGUUCGGAGAATT	RNAi

After sequence confirmation, the deduced amino acid sequence was determined. The protein function domains of CfIKK1 were predicted using the Simple Modular Architecture Research Tool (SMART, http://smart.emblheidelberg.de) and CDD/SPARCLE database (27). The protein sequences of IKKα, IKKβ, and the IKK-related gene family of different species were downloaded from the NCBI website (http://www.ncbi.nlm.nih.gov/guide/proteins/) and compared using the Clustal Omega program (https://www.ebi.ac.uk/Tools/msa/clustalo/). A phylogenetic tree of IKK proteins was constructed using the neighbor-joining method in MEGA software (v.5.05). Bootstrapping with 1000 replications was applied for evaluation of the branch nodes (http://www.megasoftware.net).

### 2.4 Analysis of *CfIKK1* mRNA Expression

To examine the *CfIKK1* gene expression levels, the quantitative reverse transcription-polymerase chain reaction (qRT-PCR) was carried out. First, total RNA was extracted from the tissue samples using TRIzol reagent as described in section 2.2. Next, reverse transcription of the RNA was performed using the PrimeScript RT Reagent Kit with gDNA Eraser (Takara, Japan). To quantify the *CfIKK1* mRNA expression levels, the qRT-PCR was employed using the SYBR Green 2× Master Mix (Takara). PCR was performed on the ABI 7500 Real-Time Thermal Cycler according to the manufacturer’s instructions (Applied Biosystems, USA), using specially designed CfIKK1-qRT-F/R primers (**Table 1**). The PCR conditions used were as follows: 2 min at 94°C, followed by 40 cycles of 30 s at 94°C, 15 s at 52°C, and 30 s at 72°C. At the end of each reaction, the melting curve was analyzed to confirm the specificity of PCR amplification. The relative level of *CfIKK1* mRNA expression was obtained with the comparative Ct method (2^–ΔΔCt^ method) (28). The housekeeping gene elongation factor-1 alpha (*EF-1α*; GenBank Accession Number: DT716075.1) was selected as the internal reference gene. For easier comparison, the *CfIKK1* expression levels of the challenged scallops were normalized to those of the PBS-injected animals. The expression data were statistically analyzed with one-way ANOVA followed by a multiple comparison or the unpaired two-tailed *t*-test using SPSS v.16.0 software. Differences with a *p-*value of less than 0.05 were considered statistically significant.

### 2.5 Cell Culture, Plasmid Construction, and Transfection

Because there is currently no mollusk cell line that allows for successful plasmid transfection, human HEK293T cells were used in this study for the protein expression assays. The cells were cultured in high-glucose Dulbecco’s modified Eagle’s medium (HyClone, USA), supplemented with 1× penicillin–streptomycin solution (Sangon, China) and 10% fetal bovine serum (Gibco, USA), at 37°C under 5% CO_2_ and subcultured every 2–3 days.

For construction of the protein-expressing plasmids, the coding sequences or the truncated fragments of different genes were amplified using Phusion High-Fidelity DNA Polymerase (Thermo Fisher Scientific, USA) with specially designed gene-specific primers (**Table 1**). In brief, for the flag-tagged plasmid construction, primers CfIKK1-FL-flag-F and CfIKK1-FL-flag-R were used to amplify the full-length *CfIKK1* sequence; CfIKK1-FL-flag-F and CfIKK1-P1-flag-R were used to amplify CfIKK1-P1, which contains the N-terminal kinase domain (KD) only; CfIKK1-FL-flag-F and CfIKK1-P2-flag-R were used to amplify CfIKK1-P2, which contains the KD and ubiquitin-like domain (ULD); and CfIKK1-P3-flag-F and CfIKK1-FL-flag-R were used to amplify CfIKK1-P3, which contains the scaffold dimerization domain (SDD) only. For the myc-tagged plasmid construction, CfMyD88-myc-F and CfMyD88-myc-R were used to amplify CfMyD88-myc; CfIKK1-FL-myc-F and CfIKK1-FL-myc-R were used to amplify the full-length *CfIKK1* sequence; CfIKK1-FL-myc-F and CfIKK1-P1-myc-R were used to amplify CfIKK1-P1; and CfIKK1-P3-myc-F and CfIKK1-FL-myc-R were used to amplify CfIKK1-P3.

After the amplification processes, the plasmid pCMV-myc (Clontech, USA) was digested with *EcoR*I (New England Biolabs, USA), and pCMS-EGFP-flag was digested with *Xho*I (New England Biolabs). The Ligation-Free Cloning System (Applied Biological Materials, Inc., Canada) was then applied to construct the expression plasmids using the digested plasmids and purified PCR products according to the manufacturer’s instructions. HEK293T cells were transfected with the protein expression plasmids using Lipofectamine 3000 reagent (Invitrogen) according to the manufacturer’s instructions.

### 2.6 Co-Immunoprecipitation Assay

Before plasmid transfection, HEK293T cells were divided into five 10-cm Petri dishes (Corning, USA) and cultured for 1 day. Thereafter, the cells were co-transfected with the myc-tagged protein expression plasmids and flag-tagged plasmids, with pCMS-flag used as a control. After 24 h, the cells were harvested using PBS, the mixture was centrifuged at low speed, the supernatant was discarded, and the pellet was lysed lysed with cell lysis buffer (Beyotime, China). Next, 20 μL of the cell lysate was put aside as an input sample, and the remaining lysate sample was added to anti-flag M2 magnetic beads (Sigma). The mixture was shaken gently at 4°C for more than 2 h, following which the magnetic beads were rinsed three times using cell lysis buffer. The 20 μL input sample and co-immunoprecipitation (co-IP) sample (the rinsed magnetic beads) were then incubated with 2× protein sodium dodecyl sulfate-polyacrylamide gel electrophoresis loading buffer (Takara) at 100°C for 5 min. The target proteins were examined with the western blot assay using the anti-myc antibody (Transgen, China) and anti-flag antibody (Sigma).

### 2.7 RNAi Assays

In order to knockdown the gene expression of *CfIKK1*, the gene-specific small-interfering RNAs targeting this gene and the negative control (NC) siRNA were designed and synthesized by Shanghai GenePharma. The negative control sequence is taken from the genome of *Caenorhabditis elegans*, and it has no homology with *Chlamys farreri* after sequence alignment. The sequences of siRNA used in this research were listed in **Table 1**. After synthesis, the siRNAs were dissolved in PBS. for CfIKK1 RNAi, each scallop was injected with approximately 11 μg of siRNA targeting CfIKK1. Negative control group was injected with NC siRNA in the same condition. The same dose of PBS was injected into scallops as a blank control. The gill tissue sampling was performed at 0 h, 6 h, 12 h, 24 h, 48 h, 72 h, 96 h, and 120 h after siRNA injection. The interference efficiency and the expression of some possible downstream genes were measured using qRT-PCR. The primers used in qRT-PCR were listed in **Table 1**.

## 3 Results

### 3.1 Coding Sequence and Deduced Amino Acids of *CfIKK1*

**Figure 1:**
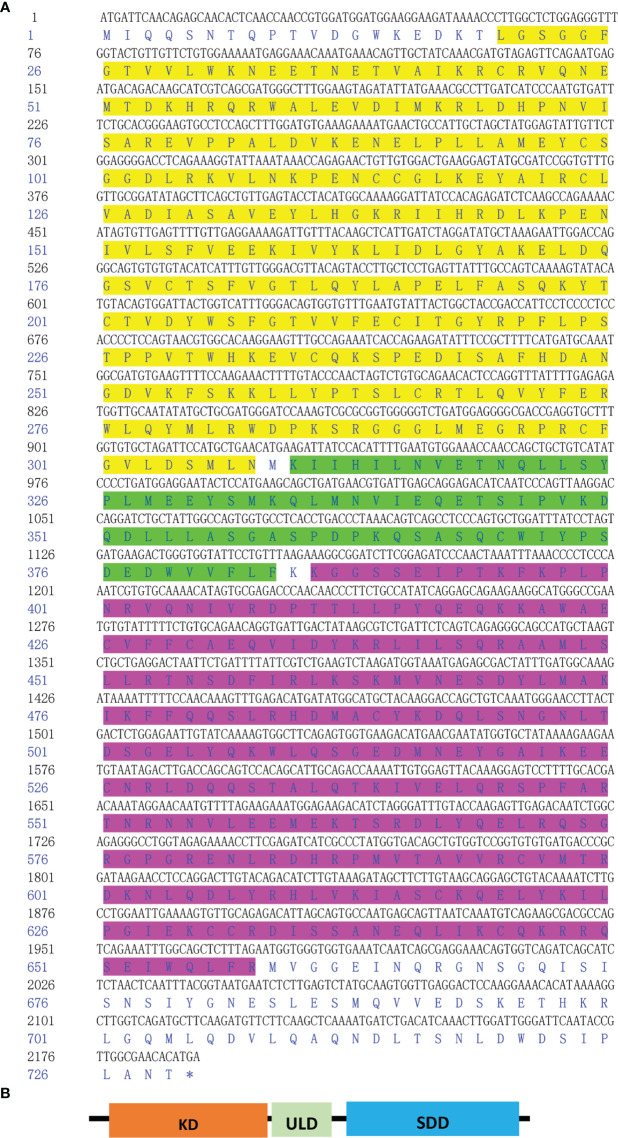
Nucleotide and deduced amino acid sequence of CfIKK1 **(A)** and schematic representation of the CfIKK1 protein **(B)**. The KD (highlighted in yellow on the sequence), ULD (green highlighted), and SDD (fuchsia highlighted) were predicted using the NCBI Conserved Domain search program (https://www.ncbi.nlm.nih.gov/Structure/cdd/wrpsb.cgi). KD, kinase domain; ULD, ubiquitin-like domain; SDD, scaffold dimerization domain.

First, primers were designed to confirm the complete coding sequence of the scallop *IKK1* gene (GenBank Accession Number: MZ727612). Because this is the first *IKK* gene cloned from *C. farreri*, we designated it *CfIKK1*. The ORF of *CfIKK1* is 2190 bp long and encodes 729 amino acids. The predicted molecular mass of the CfIKK1 protein is approximately 83.8 kDa. The deduced CfIKK1 protein contains the typical KD at the N-terminus, an ULD, and a C-terminal SDD, being similar to that in other IKK members of other species (**Figure 1**).

### 3.2 Sequence Analysis

**Figure 2:**
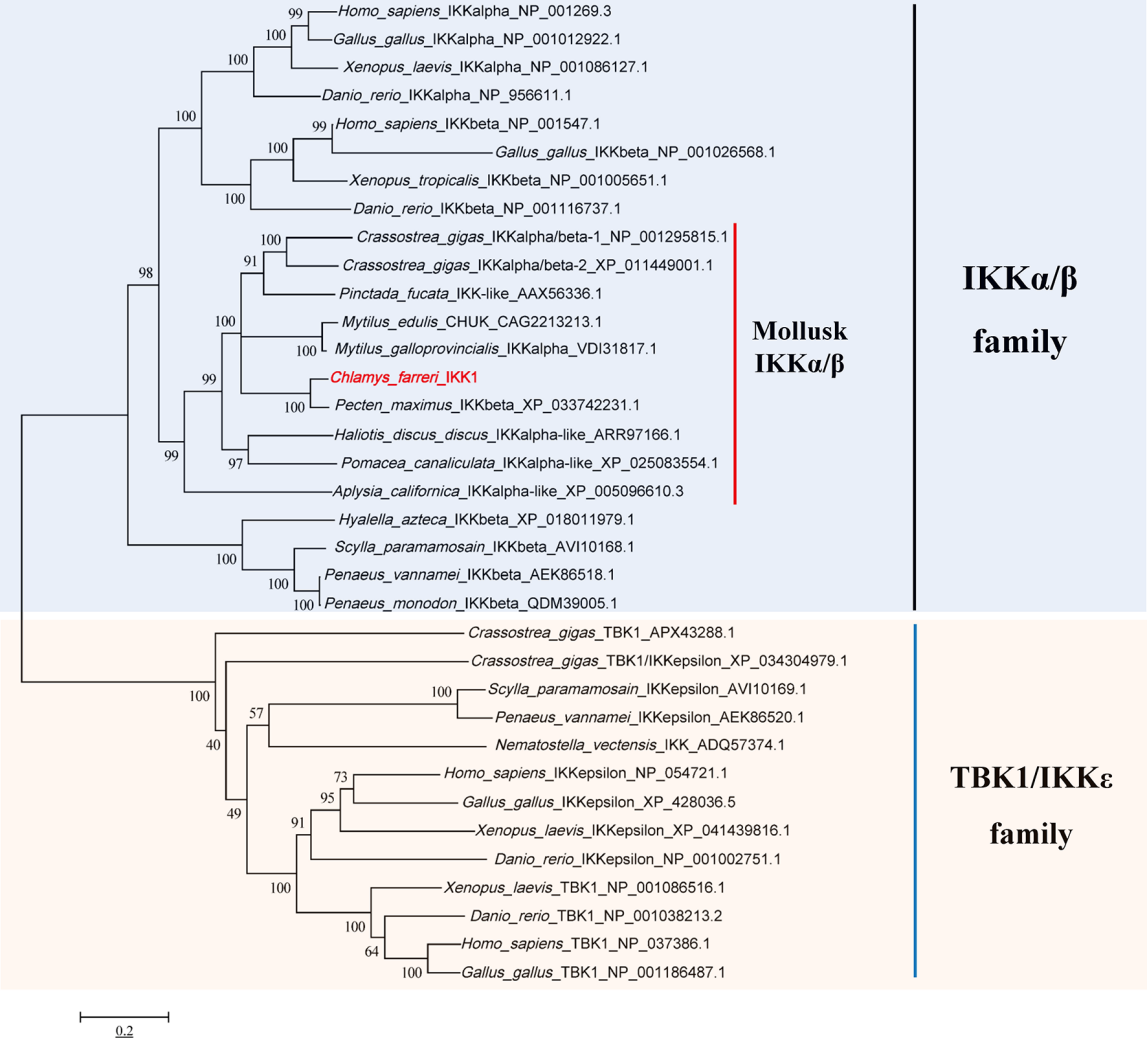
Phylogenetic tree of IKKs and IKK-related kinases from various species. The tree was constructed using the neighbor-joining method with 1000 bootstrap replications. The tree is divided into two main branches: IKKα/IKKβ family members are in the light blue section, and TBK1/IKKϵ family members are in the light brown section. The GenBank accession numbers of the protein sequences are also shown.

First, the gene sequence analysis was performed and the phylogenetic tree of the predicted IKKs and IKK-related kinases was constructed (**Figure 2**). We found that all the IKKs or IKK-related kinases used in this study could be classified into two groups: an IKKα/IKKβ group and a TBK1/IKKϵ group. CfIKK1 belonged to the IKKα (also called conserved helix-loop-helix ubiquitous kinase or Chuk) or IKKβ families. Because the IKKα and IKKβ proteins of vertebrates are clustered together in the first main branch of the phylogenetic tree, it is difficult to determine whether this scallop IKK protein is IKKα or IKKβ.

**Figure 3:**
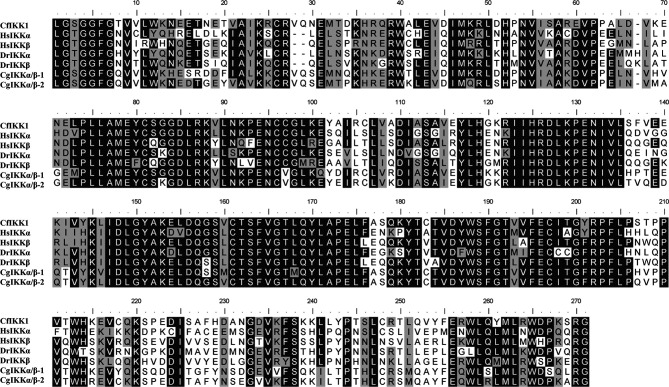
Multiple sequence alignment of the kinase domain of canonical IKK proteins from different species. Sequences with identical amino acids are shaded in black, whereas conservative amino acid substitutions are shaded in gray. The GenBank accession numbers for the sequences used are as follows: HsIKKα, NP_001269.3; HsIKKβ, NP_001547.1; DrIKKα, NP_956611.1; DrIKKβ, NP_001116737.1; CgIKKα/β-1, NP_001295815.1; CgIKKα/β-2, XP_011449001.1. Cf, *Chlamys farreri*; Hs, *Homo sapiens*; Dr, *Danio rerio*; Cg, *Crassostrea gigas*.

Next, multiple sequence alignments were performed to determine the similarity of the *CfIKK1* gene to the *IKK* genes of mollusks and other species. The protein sequence of CfIKK1 showed a relatively high identity with that of IKKα and IKKβ from *Homo sapiens* (IKKα, 41.7%; IKKβ, 41.9%, data obtained from Clustal Omega program analysis) and *Danio rerio* (IKKα, 42.1%; IKKβ, 41.2%). The amino acid sequence of CfIKK1 also shared 59.0% and 60.2% identity with oyster IKKα/β-1 and IKKα/β-2, respectively (18). However, the identity of the KD of CfIKK1 with that of other species was higher (**Figure 3**), suggesting that the KD in different species was conserved.

### 3.3 *CfIKK1* mRNA Expression Pattern

**Figure 4:**
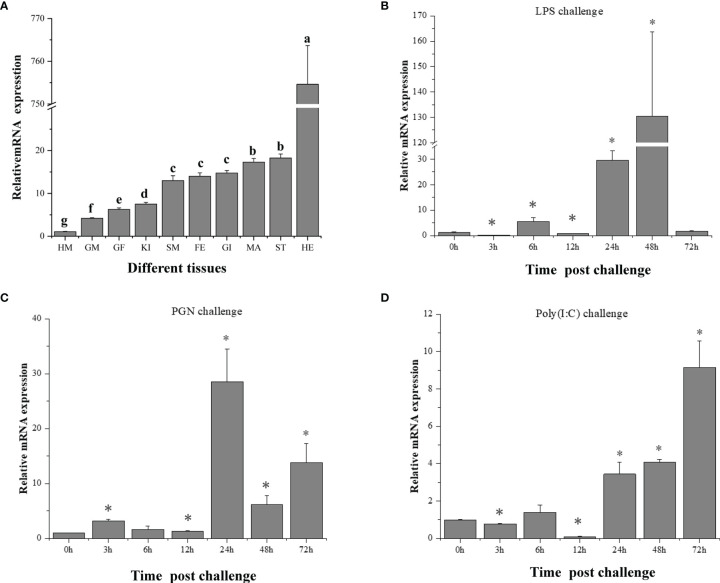
Expression of *CfIKK1* mRNA in different tissues **(A)** and after pathogen-associated molecular pattern challenges **(B–D)**, as determined using qRT-PCR. The *EF-1α* gene was used as an internal control, and hemocytes were used as a reference sample. The tissue-expression data were statistically analyzed with one-way ANOVA followed by a multiple comparison. Different letters indicate that the differences are significant (*p* < 0.05). For the challenge experiment, the different stimulants were LPS, PGN, and poly(I:C). The *CfIKK1* mRNA expression level in gill tissues was determined at 0, 3, 6, 12, 24, 48, and 72 h after challenge. *EF-1α* gene expression was used as an internal control, and time 0 h was used as a reference sample. Vertical bars represent the mean ± SD (N = 3). *p < 0.05, according to the unpaired two-tailed *t*-test. HM, hemocytes; GM, male gonad; GF, female gonad; KI, kidney; SM, smooth muscle; FE, feet; GI, gill; MA, mantle; ST, striated muscle; HE, hepatopancreas; LPS, lipopolysaccharide; PGN, peptidoglycan; poly (I:C), polyinosinic–polycytidylic acid.

To trace the expression profile and functional features of scallop CfIKK1, the tissue-specific distribution of the *CfIKK1* mRNA transcripts was evaluated using qRT-PCR. As shown in **Figure 4A**, the expression of *CfIKK1* mRNA could be detected in all tested tissues of *C. farreri*, with the level in the hepatopancreas being the highest and that in the hemocytes being relatively lower (i.e., 770-fold less than the hepatopancreas level). To confirm the role of CfIKK1 in scallop innate immunity, we studied the temporal expression profiles of *CfIKK1* in scallops challenged with bacterial and viral PAMPs. The level of *CfIKK1* mRNA expression was shown through qRT-PCR analysis to be upregulated after LPS, PGN, or poly(I:C) stimulation (**Figures 4B–D**). Regarding the temporal effects, *CfIKK1* expression was significantly upregulated at 24 and 48 h post challenge in the LPS group, greatly induced at 24 h post challenge in the PGN group, and highly upregulated at 24–72 h post challenge in the poly(I:C) group.

### 3.4 Interaction of CfIKK1 With CfMyD88

**Figure 5:**
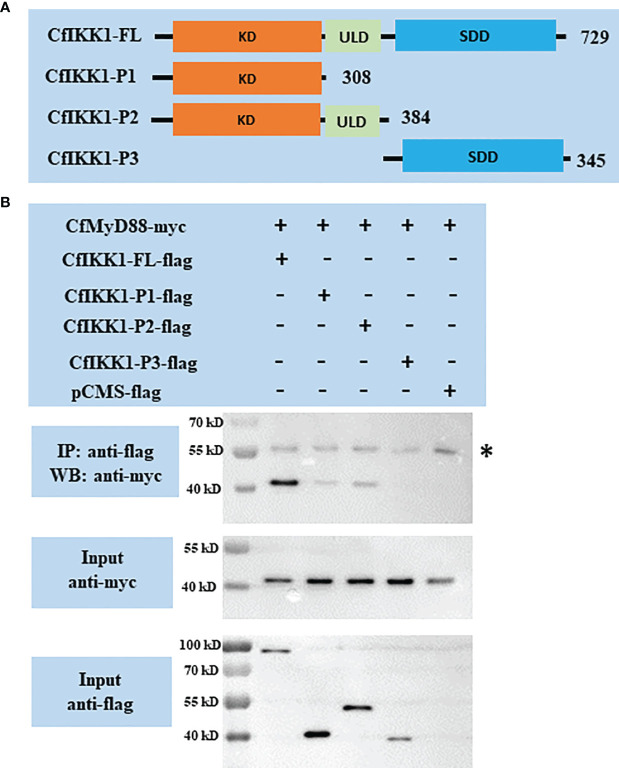
**(A)** Schematic representations of the wild-type full-length CfIKK1 protein (CfIKK1-FL) and truncated mutant CfIKK1-P1, CfIKK1-P2, and CfIKK1-P3 proteins. The protein lengths and domains are annotated. **(B)** Interaction between CfIKK1 and CfMyD88, as verified using co-immunoprecipitation (co-IP) assays. The co-IP of CfMyD88 proteins with various CfIKK1-flag proteins (CfIKK1-FL, CfIKK1-P1, CfIKK1-P2, and CfIKK1-P3) was facilitated using anti-flag M2 magnetic beads and analyzed by western blot assay using the anti-myc antibody (top). Input samples were detected using anti-myc (middle) and anti-flag antibodies (bottom), respectively, to confirm the myc- and flag-fused protein expression. CfMyD88-myc could be detected in the anti-flag immunoprecipitates of co-transfected (CfIKK1-FL, CfIKK1-P1, and CfIKK1-P2) cell extracts, indicating the interaction of CfMyD88-myc with CfIKK1-FL-flag, CfIKK1-P1-flag, and CfIKK1-P2-flag. Asterisk represents the heavy chain of mouse IgG. KD, kinase domain; SDD, scaffold dimerization domain; IP, co-immunoprecipitation assay; WB, western blot assay.

The TLR signaling pathway is a well-known host pathway for generating innate immunity against bacteria and viruses (29). In this study, we characterized the potential interaction between CfIKK1 and the scallop MyD88 protein (GenBank Accession Number: ABB76627.1), the latter of which is a key adaptor molecule of the TLR pathway. Remarkably, our co-IP and immunoblotting results showed that CfIKK1 could interact with CfMyD88 (**Figure 5**). Additionally, we constructed truncated mutants of CfIKK1 and examined the domains involved in the interaction (**Figure 5A**). As shown in **Figure 5B**, the CfIKK1-P1 mutant (containing the KD only) and the CfIKK1-P2 mutant (containing both the KD and ULD) could also interact with CfMyD88 (**Figure 5B**).

### 3.5 Homo-Interactions of CfIKK1 Molecules

**Figure 6:**
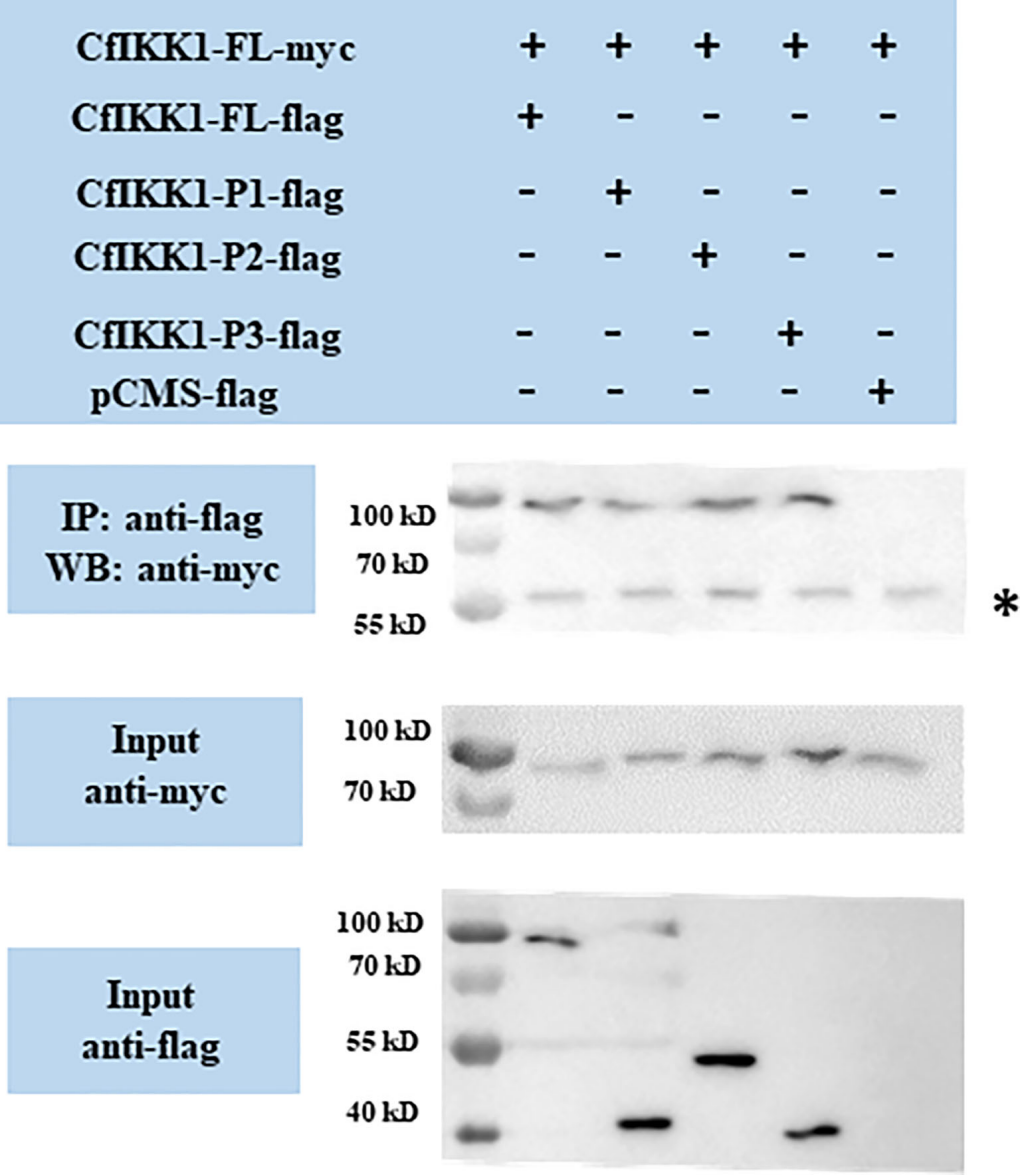
Interaction between full-length CfIKK1 proteins (CfIKK1-FL-myc) and various CfIKK1-flag proteins (CfIKK1-FL, CfIKK1-P1, CfIKK1-P2, and CfIKK1-P3), as verified using co-immunoprecipitation assays. CfIKK1-FL-myc was detected in the anti-flag immunoprecipitates of co-transfected (CfIKK1-FL, CfIKK1-P1, CfIKK1-P2, and CfIKK1-P3) cell extracts, indicating the interaction of CfIKK1-FL-myc with CfIKK1-FL-flag, CfIKK1-P1-flag, CfIKK1-P2-flag, and CfIKK1-P3-flag. Asterisk represents the heavy chain of mouse IgG. IP, co-immunoprecipitation assay; WB, western blot assay.

The dimerization and oligomerization of IKKα/IKKβ proteins and IKK complex formation are essential for the transmission of immune signals and the activation of downstream effector genes. And the self-association of IKKα/IKKβ proteins is the basis for the formation of protein complexes. Therefore, co-IP assays were employed for the confirming of CfIKK1 self-association. Firstly, we studied the interaction between the full-length CfIKK1-FL-myc and various truncated mutant flag-tagged proteins. The results also showed that CfIKK1-FL-flag, CfIKK1-P1-flag (KD only), CfIKK1-P2-flag (KD + ULD), and CfIKK1-P3-flag (SDD only) could interact with CfIKK1-FL-myc (**Figure 6**).

**Figure 7:**
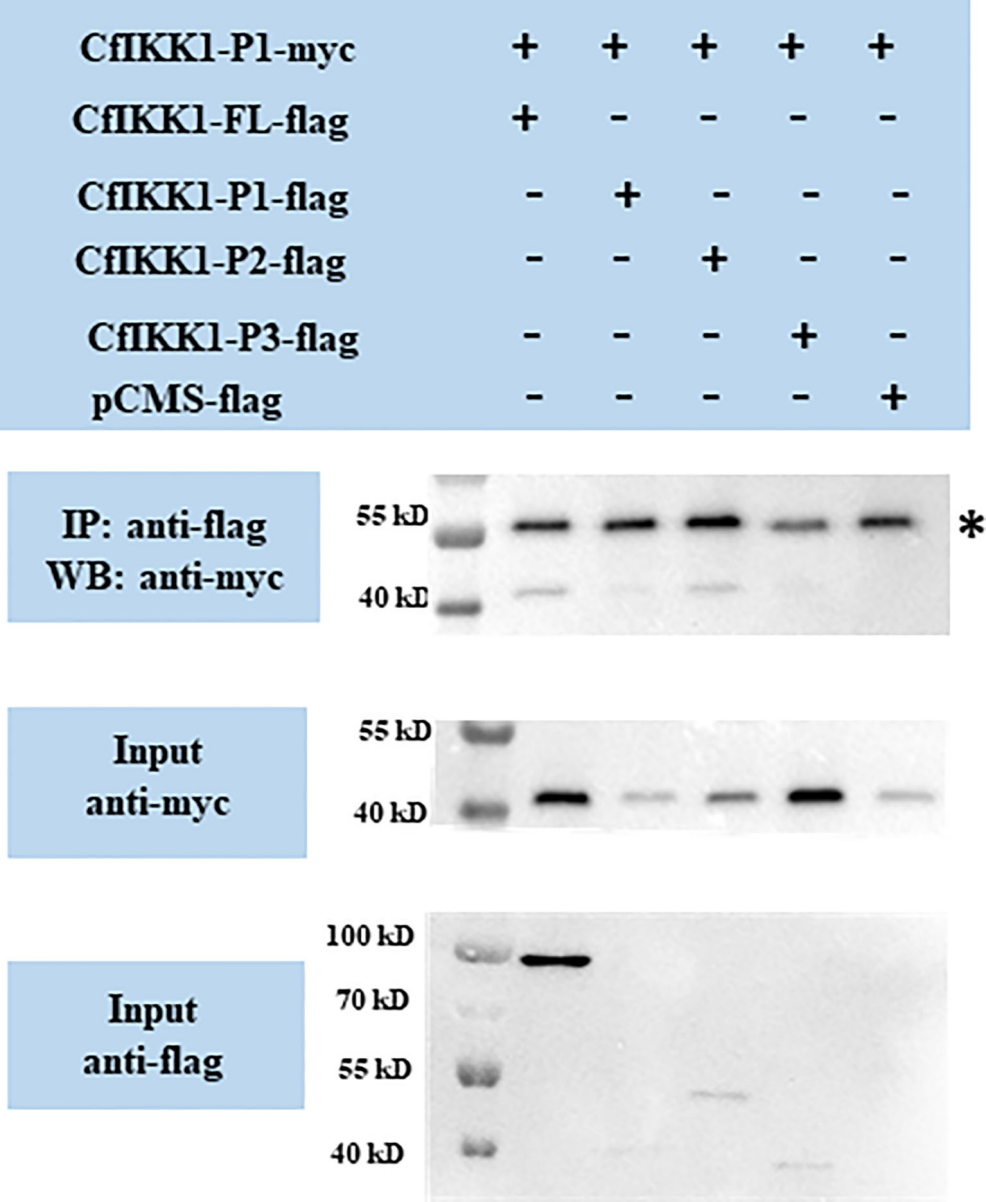
Interaction between the CfIKK1 kinase domain (CfIKK1-P1-myc) and various CfIKK1-flag proteins (CfIKK1-FL, CfIKK1-P1, CfIKK1-P2, and CfIKK1-P3), as verified using co-immunoprecipitation assays. CfIKK1-P1-myc was detected in the anti-flag immunoprecipitates of co-transfected (CfIKK1-FL, CfIKK1-P1, and CfIKK1-P2) cell extracts, indicating the interaction of CfIKK1-P1-myc with CfIKK1-FL-flag, CfIKK1-P1-flag, and CfIKK1-P2-flag. Asterisk represents the heavy chain of mouse IgG. IP, co-immunoprecipitation assay; WB, western blot assay.

Then we wondered which domain mediates the self-association of CfIKK1. CfIKK1-P1-myc expression plasmids were constructed, and the interactions between CfIKK1-P1-myc and the flag-tagged proteins were examined. The results showed that CfIKK1-FL-flag, CfIKK1-P1-flag, and CfIKK1-P2-flag could interact with CfIKK1-P1-myc (KD only) (**Figure 7**), suggesting that the KD domain plays a key role in mediating CfIKK1 self-association.

**Figure 8:**
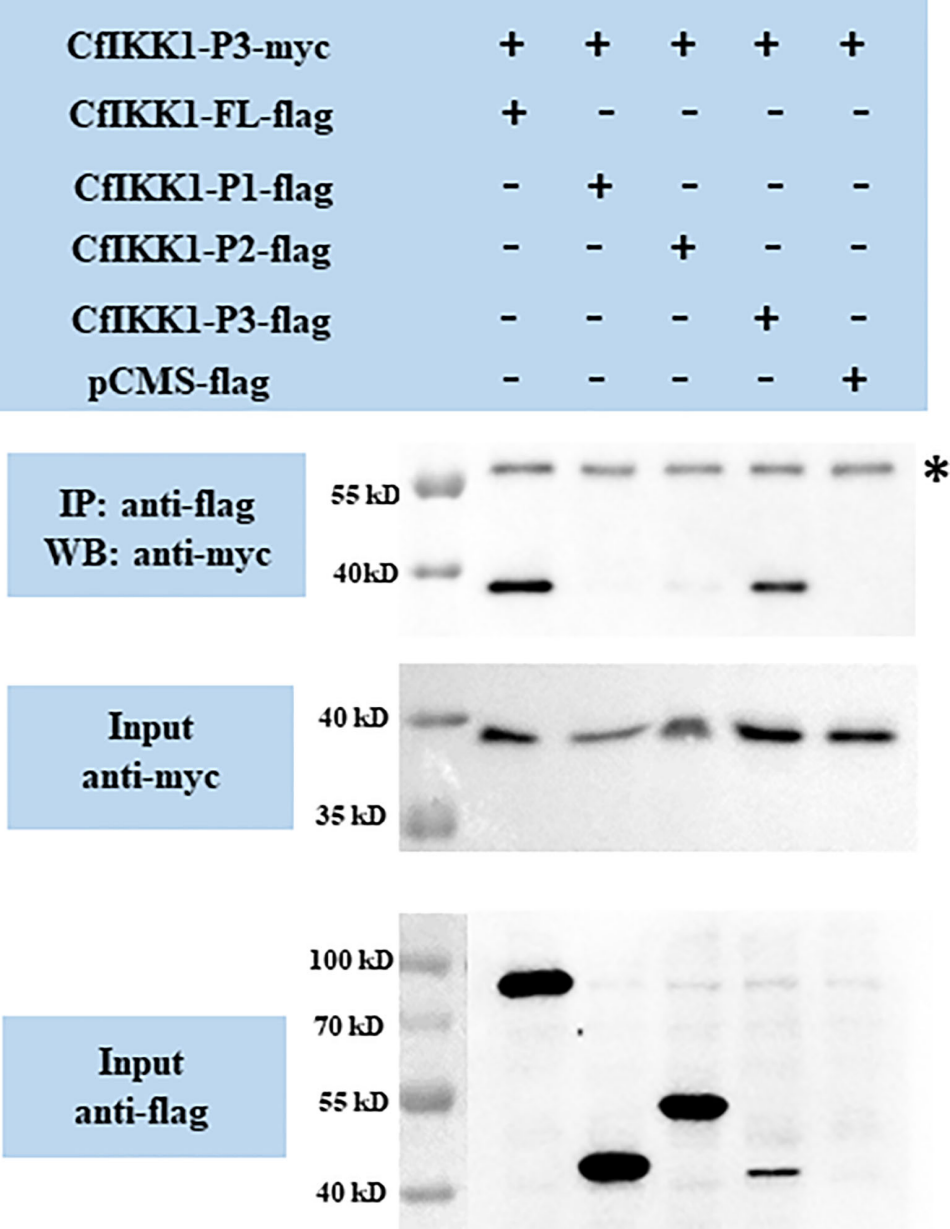
Interaction between the CfIKK1 scaffold dimerization domain (CfIKK1-P3-myc) and various CfIKK1-flag proteins (CfIKK1-FL, CfIKK1-P1, CfIKK1-P2, and CfIKK1-P3), as verified using co-immunoprecipitation assays. CfIKK1-P3-myc was detected in the anti-flag immunoprecipitates of co-transfected (CfIKK1-FL and CfIKK1-P3) cell extracts, indicating the interaction of CfIKK1-P3-myc with CfIKK1-FL-flag and CfIKK1-P3-flag. Asterisk represents the heavy chain of mouse IgG. IP, co-immunoprecipitation assay; WB, western blot assay.

CfIKK1-P3-myc expression plasmids were also constructed, and the interactions between CfIKK1-P3-myc and the flag-tagged proteins were examined. The results showed that CfIKK1-FL-flag and CfIKK1-P3-flag could interact with CfIKK1-P3-myc (SDD only) (**Figure 8**), suggesting that the SDD domain plays a key role in mediating CfIKK1 self-association.

### 3.6 The Expression of TLR Pathway Genes in Scallop During CfIKK1 RNAi

**Figure 9:**
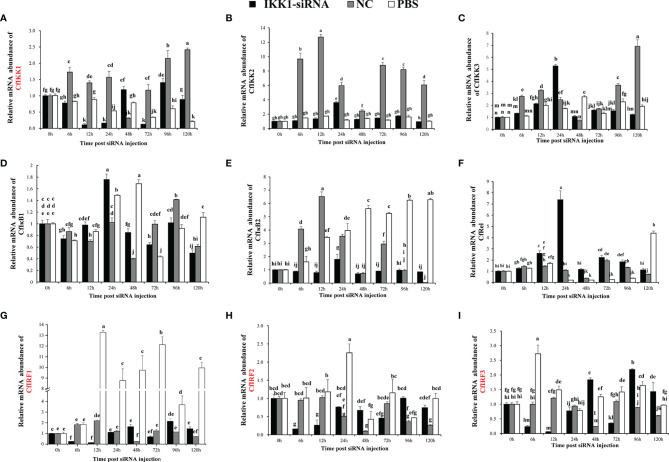
Study on the mRNA abundance of genes related to the toll-like receptor (TLR) signaling pathway in *C. farreri* during CfIKK1 RNAi: **(A)** *CfIKK1*, **(B)** *CfIKK2*, **(C)** *CfIKK3*, **(D)** *CfIκB1*, **(E)** *CfIκB2*, **(F)** *CfRel*, **(G)** *CfIRF1*, **(H)** *CfIRF2*, **(I)** *CfIRF3*. *CfIKK1*-siRNA was designed based on the gene sequence of *CfIKK1*. And the sequence of negative control (NC) siRNA is taken from the genome of *Caenorhabditis elegans*, and it has no homology with *Chlamys farreri* after sequence alignment. Gill tissue was sampled for total RNA extraction and gene expression studies. The *EF-1α* gene was used as an internal control to calibrate the gene expression of all samples. Each vertical bar represents the mean ± SD (n = 3). Different letters indicate that the differences are significant (*p* < 0.05). The genes whose expression was significantly decreased after *CfIKK1*-RNAi were marked in red.

The effect of siRNA-mediated RNAi on scallop *CfIKK1* gene was examined by real-time qRT-PCR. In *CfIKK1*-RNAi assay, the expression level of *CfIKK1* gene decreased at 6 hours after siRNA injection and remained at a relatively low level (lower than 0.3 times the normal expression level) for 12 h and 24 hours (**Figure 9A**). In the results, we found that the expression of *CfIKK2* (The sequence has not been submitted) and *CfIKK3* (The sequence has not been submitted) did not change significantly. And the expression of *CfIκB1* (The sequence has not been submitted) was decreased slightly at 6 h after *CfIKK1* interference, *CfIκB2* (DQ852572.2) expression was relatively stable and *CfRel* (MW805350) expression was up-regulated in 0 h – 24 h after siRNA injection. Interestingly, when the expression level of *CfIKK1* down-regulated for 6 h – 24 h, the expression level of *CfIRF1* (The sequence has not been submitted), *CfIRF2* (The sequence has not been submitted), and *CfIRF3* (The sequence has not been submitted) showed a significant decrease. At the same time, we also noticed that in the animals of negative control group which were injected with NC siRNA, the expression of some genes such as *CfIKK1*, *CfIKK2*, *CfIKK3*, and *CfIκB2* was also obviously induced (**Figure 9**).

## 4 Discussion

Aside from its ecological and evolutionary importance, the Zhikong scallop (*C. farreri*) has high commercial value in China (21, 30). However, in recent years, bacteria and viruses have caused the mass mortality of this species, resulting in major economic losses to the scallop farming industry (22, 23). As an invertebrate, the scallop lacks an adaptive immune system and relies on innate immunity alone for defense against infectious pathogens. Therefore, there is a need to elucidate the innate antiviral or antibacterial immune mechanisms of the scallop. In this study, a new *IKK* gene was verified in *C. farreri* for the first time and was designated *CfIKK1*. This gene encodes a putative protein of 729 amino acids, with a predicted molecular mass of approximately 83.8 kDa. Protein domain prediction revealed that the CfIKK1 protein was a typical IKK family member, containing the N-terminal KD and ULD and C-terminal SDD (**Figure 1**). In our previous study, we showed that the genome of the Pacific oyster (*Crassostrea gigas*) carries several *IKK* and IKK-related kinase genes (18). The expansion of their number may give these genes more sophisticated regulatory mechanisms and reveals that the molluscan IKK proteins may have divergent functions under PAMP stimulation.

After the sequence confirmation, the phylogenetic relationship of IKK proteins of different species was analyzed. The IKKα and IKKβ proteins of vertebrates were clustered together in the first branch of the phylogenetic tree, revealing that the *IKKα* and *IKKβ* genes of vertebrates may be derived from the same ancestral gene after duplication and functional differentiation. Because of this, it was difficult for us to distinguish whether *CfIKK1* is *IKKα* or *IKKβ*. As a member of the invertebrate family of canonical IKKs, CfIKK1—with its N-terminal KD—showed a high degree of sequence conservation with IKKs of other species (**Figure 3**). Therefore, the function of the *CfIKK1* gene may also be conserved in relation to that in vertebrates.

Unveiling the tissue-specific expression patterns of genes is often useful for determining their potential functions. As shown in **Figure 4A**, *CfIKK1* mRNA was detected in all the tissues tested, with the expression level in the hepatopancreas being much higher than that in the other tissues. The ubiquitous expression of *CfIKK1* mRNA indicates that the protein could be essential to most of the basic physiological functions of *C. farreri*. The hepatopancreas is thought to be an important organ for synthesizing proteins involved in immune defense (31), and many scallop immune-related genes are highly expressed in this tissue (32, 33). Similarly, many immune-related genes have been found to be highly expressed in the digestive glands of oysters (34, 35). Therefore, the abundance of *CfIKK1* mRNA transcripts in the hepatopancreas suggests the important role that CfIKK1 may play in the *C. farreri* immune response to external pathogens.

To elucidate the role that CfIKK1 plays in the scallop immune responses, the mRNA expression profiles were examined following stimulation of the individuals with LPS, PGN (a bacteria-related PAMP), or poly(I:C) (a virus-related PAMP). Overall, *CfIKK1* mRNA expression in the scallop gills was induced to a significant extent by LPS, PGN, and poly(I:C) challenges, indicating that CfIKK1 likely participates in the scallop’s innate immune defense against bacterial and viral infections. And the induced expression by bacterial and viral PAMP challenge suggests the multifunctionality of this kinase in scallop innate immunity. At the same time, we also observed that although these PAMP challenge significantly induced the expression of *CfIKK1*, the timing of the onset of IKK-induced expression and the peak expression were different. After LPS challenge, the expression of *CfIKK1* peaked at 48 h, PGN 24 h, and poly(I:C) 72 h, indicating that scallops respond to different PAMP stimuli and have different mechanisms to initiate *CfIKK1* expression. It also shows the complexity of the regulatory mechanisms of scallops in response to pathogenic stimulation.

It is well known that the mammalian TLR signaling pathway plays a crucial role in defending against pathogenic microbial infection through the activation of NF-κB and IRF3/IRF7 (36, 37). Moreover, the IKK proteins are important signaling molecules in the TLR pathways (4, 29). It was previously suggested that there may be a primitive TLR signaling pathway in *C. farreri* (38), and the most crucial adaptor protein of TLR signaling, MyD88, was also cloned from this scallop species (39). However, the interacting proteins and the roles that scallop IKK proteins play in the TLR signaling pathways are still largely unknown. In this study, our co-IP and western blotting results showed that CfMyD88 could interact with CfIKK1 (**Figure 5**), suggesting that CfIKK1 may be involved in the scallop TLR signaling pathway *via* binding MyD88 adaptor. Additionally, the truncated mutants of CfIKK1 containing the KD only (CfIKK1-P1) or KD + ULD (CfIKK1-P2) also interacted with CfMyD88, revealing that the KD alone would be sufficient for interacting with adaptor and signal transduction molecules.

IKK complex formation *via* the dimerization and oligomerization of IKK proteins is considered to be important for pathway signaling and downstream target protein activation (4). In this study, co-IP assays were conducted for the validation of CfIKK1 self-association. The results showed that both the KD and SDD would be crucial for the dimerization or even oligomerization of CfIKK1. Although these two function domains play an important role in mediating the self-association of CfIKK1, we speculate that it may function at different times of IKK complex formation. It is possible that the KD domain plays a key role at the homodimerization of this protein, while SDD domain functions when dimers combine with each other to form oligomers. Of course, we need more in-depth experiments to verify the detailed IKK complex formation and IRF regulation mechanism.

Since scallop CfIKK1 could bind to CfMyD88 and may participate in the TLR signaling pathway, and CfIKK1 protein could form homodimers and even oligomers, which could be important in the IKK complex formation. However, little is known about the downstream genes of scallop IKK1. Therefore, in this study, expression of CfIKK1 was knocked down with the help of RNAi experiments, and then the expression of other genes which may be involved in the scallop TLR pathway was studied through qRT-PCR experiments (**Figure 9**) (38, 40). It turned out that when the expression of *CfIKK1* was suppressed, the expression of the three *IRF* genes of *C. farreri* was significantly down-regulated, suggesting that IKK1 may play a key role in regulating of IRFs activation and function. And interestingly, the expression of the NF-κB family gene (*CfRel*) of *C. farreri* was significantly up-regulated at 12h and 24h after IKK1-siRNA injection. What needs to be mentioned is that the IKKα/IKKβ proteins of vertebrates mainly play a key role in regulating NF-κB activation (4, 6). While in scallops, IKK1, which belongs to IKKα/IKKβ family showed a tighter regulatory relationship with the transcription factor IRFs. And in NF-κB activation process, IKK1 appears to act as a negative regulator. This may also be an indication of the functional differences of IKKα/IKKβ in vertebrates and invertebrates. Therefore, innate immune signal transduction and activation regulation mechanisms in invertebrates need more in-depth research. It should be mentioned that the siRNA of negative control (NC) was also a kind of exogenous nucleic acid for *Chlamys farreri*, so it may act as a foreign PAMP to cause scallop immune response and induce the expression of immune-related genes. This may be the reason why the expression of *CfIKK1*, *CfIKK2*, *CfIKK3*, and *CfIκB2* was also obviously induced in the NC group.

**Figure 10:**
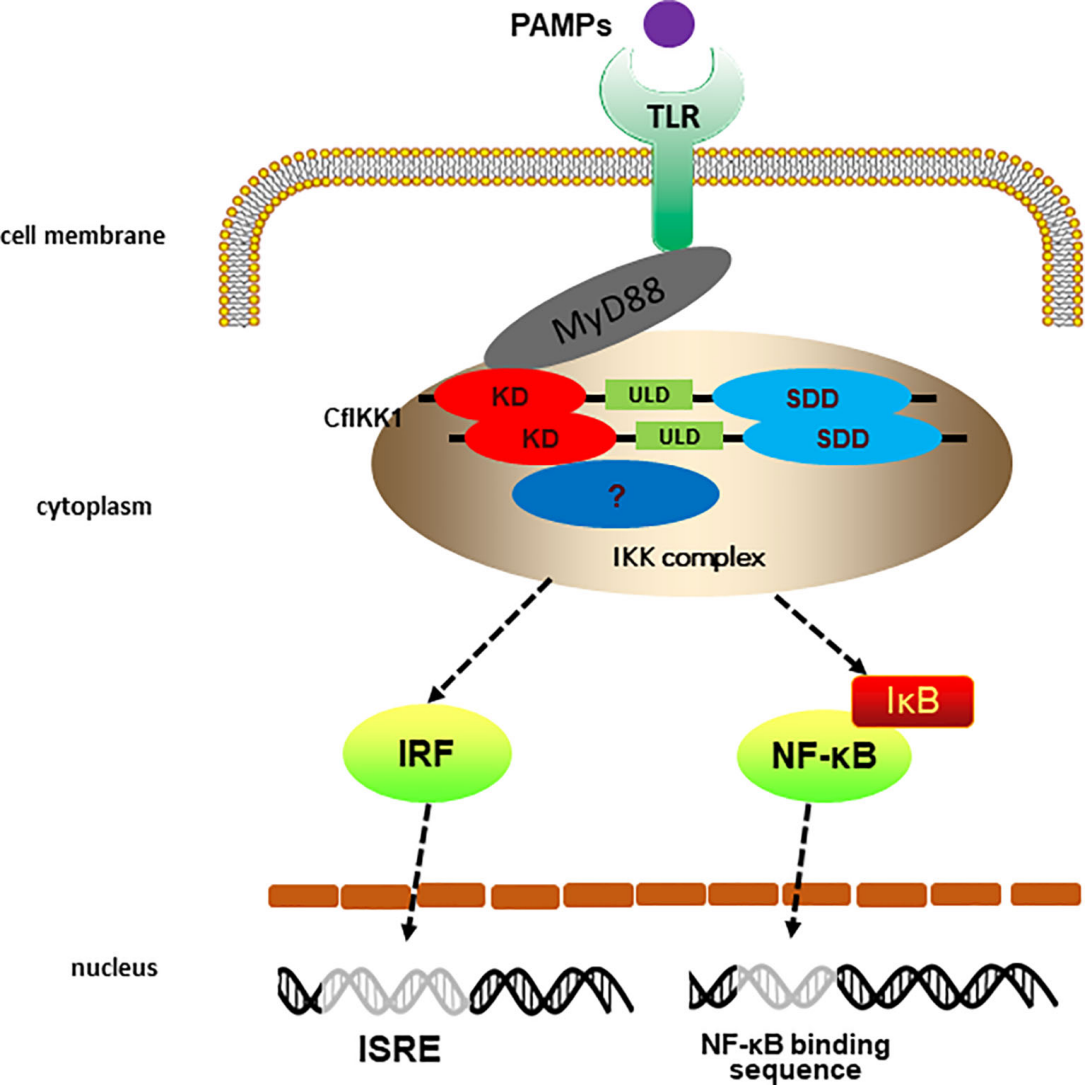
Predicted diagram of the scallop the toll-like receptor (TLR) signaling that CfIKK1 mediates. The PAMPs challenge activates the scallop pattern recognition receptors TLR and then scallop TLR binds the corresponding adaptor molecule CfMyD88. Next, CfMyD88 recruit CfIKK1 for signaling. The dimerization and oligomerization of CfIKK1 may be a crucial step of IKK complex formation. Then the IKK complex activates scallop interferon regulatory factors (IRFs) and nuclear factor-κB (NF-κB).

Combined with previous studies (38), we preliminarily outlined the TLR signaling mediated by scallop IKK1 (**Figure 10**). Briefly, the stimulation of external pathogenic microorganisms PAMPs activate the pattern recognition receptor TLR of scallops, and then the TLR uses its adaptor protein CfMyD88 to further recruit CfIKK1 for signal transmission. After that, CfIKK1 dimerizes or polymerizes, and forms an IKK complex with the help of other proteins. The IKK complex will further activate downstream transcription factors such as IRFs. However, further study is needed to elucidate the specific details of such signal transducer and activation of transcription factors.

In conclusion, the scallop gene *CfIKK1* was identified from the Zhikong scallop (*C. farreri*) for the first time. CfIKK1 belongs to the invertebrate IKKα/IKKβ family. *CfIKK1* mRNA was expressed ubiquitously in the scallop body, particularly in the hepatopancreas, and the gene responded to LPS, PGN, and poly(I:C) stimulation. CfIKK1 could bind to CfMyD88 *via* its KD domain, indicating its crucial role in the scallop TLR signaling pathway. Moreover, CfIKK1 could form homodimers or homo-oligomers *via* KD and SDD, which may be a key step in the activation of this signal transduction molecule. Meanwhile, with the help of RNAi experiments, we found that there may be a close regulatory relationship between scallop IKK1 and IRF, reflecting the differences in IRF activation mechanisms between vertebrates and invertebrates. Taken together, these results support our hypothesis that CfIKK1 is an important signal transduction molecule in the scallop TLR signaling pathway and participates in the regulation of innate immunity in this species. The results of our study provide a theoretical basis for further in-depth research on the functions of the genes in the scallop innate immune pathways.

## Data Availability Statement

The original contributions presented in the study are included in the article/supplementary material. Further inquiries can be directed to the corresponding author.

## Author Contributions

BH and XTW conceived and designed the experiments. LL, WL, FL, BH, NF, QL, XMW, YZ, XS, JD, XNW, JM, and JC performed the experiments. LW, YL, MZ, and YQ analyzed the data. BH, LL, and XTW wrote the manuscript. All authors read and approved the final manuscript.

## Funding

This research was supported by the Agricultural Variety Improvement Project of Shandong Province (No. 2019LZGC020), the National Key R&D Program of China (No. 2018YFD0901400), the National Natural Science Foundation of China (Nos. 31802328, 41876193, 41906088 and 42076088), the Special Funds for Taishan Scholars Project of Shandong Province, China (No. tsqn201812094), the Shandong Provincial Natural Science Foundation, China (No. ZR2019MC002), the Modern Agricultural Industry Technology System of Shandong Province, China (SDAIT-14-03), and the Plan of Excellent Youth Innovation Team of Colleges and Universities in Shandong Province, China (2019KJF004).

## Conflict of Interest

The authors declare that the research was conducted in the absence of any commercial or financial relationships that could be construed as a potential conflict of interest.

## Publisher’s Note

All claims expressed in this article are solely those of the authors and do not necessarily represent those of their affiliated organizations, or those of the publisher, the editors and the reviewers. Any product that may be evaluated in this article, or claim that may be made by its manufacturer, is not guaranteed or endorsed by the publisher.
